# Risk factors and early outcomes associated with prolonged pleural effusion/chylothorax after paediatric cardiac surgery

**DOI:** 10.1093/ejcts/ezae363

**Published:** 2024-10-07

**Authors:** Dan M Dorobantu, Peter Davis, Katherine Brown, Deborah Ridout, Paul Wellman, Jane Cassidy, Christina Pagel, Warren Rodrigues, Serban C Stoica

**Affiliations:** Children’s Health and Exercise Research Center, University of Exeter, Exeter, UK; Cardiology and Intensive Care Departments, Bristol Royal Hospital for Children and the Heart Institute, Bristol, UK; Cardiology and Intensive Care Departments, Bristol Royal Hospital for Children and the Heart Institute, Bristol, UK; Intensive Care Unit Department, Great Ormond Street Hospital for Children, London, UK; Population, Policy and Practice Programme, University College London, London, UK; Intensive Care Unit Department, Evelina London Children’s Hospital, London, UK; Intensive Care Unit Department, Birmingham Children’s Hospital, Birmingham, UK; Population, Policy and Practice Programme, University College London, London, UK; Intensive Care Unit Department, Great Ormond Street Hospital for Children, London, UK; Cardiology and Intensive Care Departments, Bristol Royal Hospital for Children and the Heart Institute, Bristol, UK

**Keywords:** Pleural effusion, Chylothorax, Morbidity, Prospective study, Congenital heart disease, Paediatric cardiac surgery

## Abstract

**OBJECTIVES:**

Prolonged pleural effusion/chylothorax (PPE/C) is a less investigated complication following paediatric cardiac surgery, and its true incidence, risk factors and impact on postoperative outcomes are not well described. We aim to address these gaps in knowledge using data from a prospective, multicentre study.

**METHODS:**

Data on 9 post-operative morbidities (unplanned reinterventions, extracorporeal life support, necrotising enterocolitis, PPE/C, renal replacement therapy, major adverse events, acute neurological events, feeding issues and postsurgical infection) were prospectively collected at 5 UK centres between 2015 and 2017, following paediatric cardiac surgery. Incidence of PPE/C, associations with procedure types, and risk factors were described. Mortality (30-day and 6-month) and hospital length of stay (HLoS) were compared between those with isolated PPE/C, single non-PPE/C morbidity, no morbidity, multimorbidity PPE/C and non-PPE/C multimorbidity.

**RESULTS:**

A total of 3090 procedures (2861 patients) were included (median age, 228 days). There were 202 PPE/C (incidence of 6.5%), occurring at a median of 6 days postoperatively (interquartile range: 3–10). PPE/C was associated with excess early mortality only when complicating scenarios where at least 2 other post-operative morbidities occurred. On average PPE/C is associated with 8 more HLoS days, but the relative impact is greatest when comparing isolated PPE/C with no morbidity (*P* < 0.001), whereas in multimorbidity scenarios, PPE/C does not significantly contribute to an increase of HLoS.

**CONCLUSIONS:**

Addition of PPE/C increases mortality but not HLoS in multimorbidity and HLoS only in single morbidity scenarios. This reinforces the important role of prevention, early detection and management of PPE/C in complex situations.

## Graphical Abstract

**Figure:**
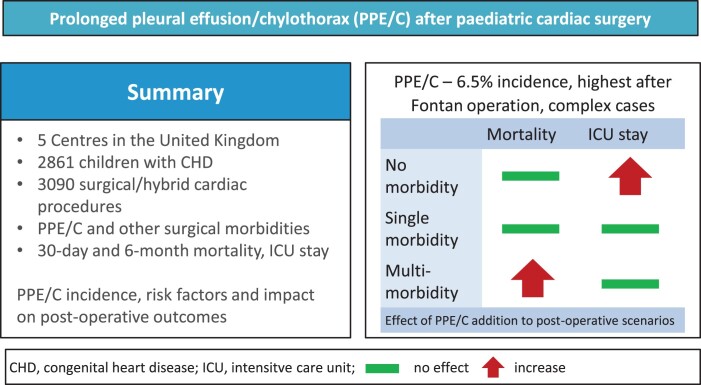

## INTRODUCTION

Prolonged pleural effusion or chylothorax (PPE/C) is one of the major complications seen after paediatric cardiac surgery, and is associated with longer hospitalization, patient morbidity and potentially increased mortality [1–3]. There are few modern reports on the true incidence of PPE/C in children undergoing surgery for congenital heart disease (CHD), with estimates from 1.3% to 9.2% [4–10], but with greatly different definitions and patient groups. More data is available specifically for patients undergoing the Fontan operation, where the incidence of chylothorax reported to be up to 45% [11], with few reports on incidence after other specific procedures.

Evaluating the risk factors for PPE/C and impact on outcome is difficult, due to how its causes are multifactorial, and connected to other postoperative complications [1]. More complex procedures, younger age, more severely ill patients were all proposed as risk factors for PPE/C [4]. Whether PPE/C occurrence increases patient morbidity and mortality, independent of these other associated complications is not known.

Data on how PPE/C affects early and late morbidity and mortality are scarce, and conflicting. A large registry report from North America showed that even after adjusting to patient and procedure factors, chylothorax was still associated with longer intensive care unit stay and higher early mortality [4]. On the other hand, a different large single-centre study found no excess adjusted mortality associated with chylothorax [7]. In patients with univentricular circulation, postoperative chylothorax was found to be a predictor for medium-term Fontan failure [12], but this is likely confounded by haemodynamic characteristics.

These differences in observed added mortality in the presence of PPE/C, could very likely be due to the unaccounted impact of other postoperative events, and this hypothesis is supported by earlier studies associating both PPE/C and mortality to the need for extracorporeal life support (ECLS) [3], and our group’s earlier report of multimorbidity data, showing a significant degree of overlap between major post-surgical morbidities, including PPE/C [10].

The current study aims to: (i) report the incidence of PPE/C in an unselected paediatric population undergoing cardiac surgery, as well as by specific procedures; (ii) explore predictors of PPE/C in this setting; (iii) evaluate the relationship between PPE/C, other post-operative morbidities and early outcomes. This is a secondary, exploratory, rather than hypothesis-based analysis, focused on PPE/C, from a large multicentre prospective study collecting information on 9 major postoperative morbidities, described previously [10].

## PATIENTS AND METHODS

### Study design and dataset

Study data collection was described previously [10]. This study was approved by the North of Scotland Research Ethics Committee (trial #18/NS/0106). Briefly, the dataset included all consecutive patients aged 0–17 years undergoing cardiac surgery or hybrid procedures at 5 participating centres between October 2015 and June 2017. Nine morbidities were recorded, as described before [13]. These were: unplanned reinterventions, ECLS, necrotising enterocolitis, PPE/C, renal replacement therapy, major adverse events, acute neurological events, feeding issues and postsurgical infection (for definition see Supplementary Material, Table S1). The anonymised cardiac surgical, diagnostic and risk factor data were collected from the National Congenital Heart Disease Audit (NCHDA) [10, 14].

### Definition of reintervention and mortality outcomes

#### Risk factor and variable groups

Clinical data collected from patient records and the NCHDA are detailed in the Supplementary Material and were described previously [10, 15]. Of interest for the current analysis, pleural effusion or chylothorax was defined as either a chylous pleural effusion or significant chylous pericardial effusion or significant chylous ascites or a prolonged nonchylous effusion. Prolonged pleural effusion was defined as necessitating thoracic drainage >10 days (i.e. effusion noted in patient records beyond the 10th day postoperatively). Age at PPE/C was based on the time PPE/C was first mentioned. Chylothorax could be noted at any point during the hospital stay, regardless of duration, if documented through standard-of-care testing (Supplementary Material, Table S1). There were various possible scenarios based on timing of effusion to be classified as PPE/C, detailed in the Supplementary Material, Fig. S1.

Accurate chylous versus non-chylous classification could not be obtained throughout, without additional testing, so this was not used in this analysis. PPE/C severity was defined based on volume of fluid loss as: mild—always <3 ml/kg/h or average always <72 ml/kg in 24 h; moderate—3–10 ml/kg/h or average 73–240 ml/kg in 24 h; and severe—>10 ml/kg/h or average >240 ml/kg/24 h.

The Partial Risk Adjustment in Surgery Score (PRAiS) version 2 was calculated for each index procedure [16]. The PRAiS score was developed and validated using NCHDA data [16] and it offers a predicted probability of death at 30 days (expressed as %) based on several parameters, including diagnosis group, procedure type and comorbidities. In 9 patients where the procedure weight was improbable for age PRAiS was not calculated and age was marked as missing (0.3%) and not imputed. For age and severity of PPE/C complete case analysis was used. Other variables had no missing values.

Mortality was ascertained at 30 days and 6 months after the index procedure, and hospital length of stay (HLoS) was calculated based on age at admission and discharge for the index hospitalization.

### Statistical analysis

Frequencies are presented as numbers and percentages and all continuous variables as median (interquartile range). The Fisher’s test was used for comparisons of proportions and the Mann–Whitney *U*-test was used for comparisons of continuous variables.

There were several statistical analysis streams summarized below and detailed in the Supplementary Methodology section:

1. *Identifying factors associated with PPE/C and moderate or severe PPE/C:* we performed univariable and multivariable mixed logistic regression models analysis with PPE/C event (and within the PPE/C subgroup, moderate or severe grading) as the dependent variable and centre as a random effect factor (intercept). The final multivariable model includes all independently significant factors (*P* < 0.05). Only one factor was associated with severity of PPE/C so only univariable analysis was included. Candidate variable list, methodological approach and univariable analysis results are shown in Supplementary Material, Tables S2 and S3.
2. *Investigating the 30-day and 6-moth mortality attributable to the presence of PPE/C:* to account for the interaction between PPE/C presence and the number of associated morbidities, we defined an ‘associated postoperative morbidities’ variable to be: ‘0’ if no morbidities, ‘1’ if one non-PPE morbidity, ‘2’ if 2 or more, non-PPE, non-ECLS morbidities. ECLS was excluded as an associated morbidity for this comparison, due to the specific high impact on mortality and propensity for other major adverse events [10], and described as a standalone group. This resulted in 7 groups, defined in detail in Supplementary Material, Table S4. To account for the survivor and immortal time biases (potentially affecting *n* = 19 cases), a survival analysis with time-varying covariates in multiple-record data approach was used at each PPE/C or associated morbidity group status change. In some patients, time to PPE/C (11%) or first non-PPE/C morbidity (15%) was not documented, and status change was attributed at first day post-operatively. For 30-day mortality, centre and associated morbidity group stratified aggregate log-rank test statistics were reported (no events in some sub-strata). For 6-month mortality time varying PPE/C status and associated postoperative morbidities were included as a full factorial interaction term in a multiple-record Cox regression, stratified by centre.
3. *Investigating the added effect of PPE/C on HLoS:* the same PPE/C and associated morbidity-defined groups were used as above, as a fixed interaction term within a mixed linear regression model, with centre as a random effects term (intercept). We performed the analysis on the whole cohort and repeated it on hospital survivors to account for survivor bias, obtaining similar results.

When performing pairwise post-hoc comparisons within associated morbidity groups, a Bonferroni adjustment was used. These analyses were repeated including PRAiS score as a covariate, obtaining similar results. Further details on statistical methods are presented in Supplementary Methods.

All statistical analyses were done using the Stata/SE 16.0 package (StataCorp LLC, College Station, TX, USA).

## RESULTS

A total of 3090 surgical 30-day episodes (with index procedures) pertaining to 2861 patients were included, where 2648 patients had one, 197 had 2 and 16 had 3 procedures. In the whole cohort, early (30-day) mortality was 1.3%, 6-month mortality was 3% and the PRAiS score was 1.3%, as previously reported [15].

A total of 202 PPE/C were reported in *n* = 200 patients, resulting in a PPE/C incidence of 6.5%. Of all PPE/C cases, *n* = 111 were isolated PPE/C (no other postoperative morbidities) and *n* = 91 were multimorbidity PPE/C (other postoperative morbidities occurring during the same period, of which 18 were with ECLS). All demographic, clinical and procedural data are detailed in Table 1, showing patient or procedure factors associated with PPE/C.

**Table 1:** Demographic, clinical and procedural data in procedures with and without a PPE/C

	With PPE/C	Without PPE/C	Total	*P*-value
	*n* = 202	*n* = 2888	*n* = 3090	
Demographic				
Age (years), median (IQR)	0.7 (0.2–4.2)	0.6 (0.2–3.7)	0.6 (0.2–3.8)	0.8
Male, *n* (%)	120 (59.4)	1551 (53.7)	1671 (54.1)	0.1
Neonate, *n* (%)	38 (18.8)	490 (17)	528 (17.1)	0.5
Weight (kg), median (IQR)^a^	7.6 (4.6–15.3)	6.9 (4–15)	6.9 (4.1–15)	0.6
Low weight for age, *n* (%)^a^	64 (31.7)	984 (34.2)	1048 (34)	0.5
Preoperative clinical				
Diagnosis complexity class, *n* (%)				
A (most severe/complex)	36 (17.8)	241 (8.4)	277 (9)	<0.0001
B	37 (18.3)	304 (10.5)	341 (11)	
C	28 (13.9)	297 (10.3)	325 (10.5)	
D	78 (38.6)	945 (32.7)	1023 (33.1)	
E (least severe/complex)	23 (11.4)	1101 (38.1)	1124 (36.4)	
Diagnoses with >50 patients, *n* (%)				
VSD	3 (1.5)	332 (11.5)	335 (10.8)	<0.0001
ToF/DORV	23 (11.4)	288 (10)	311 (10.1)	
CAVSD	23 (11.4)	271 (9.4)	294 (9.5)	
Aortic arch obstruction + septal defect	9 (4.5)	195 (6.8)	204 (6.6)	
Hypoplastic left heart syndrome	29 (14.4)	168 (5.8)	197 (6.4)	
TGA-VSD/DORV-TGA	18 (8.9)	174 (6)	192 (6.2)	
Functionally UVH	26 (12.9)	161 (5.6)	187 (6.1)	
Atrial septal defect	0 (0)	185 (6.4)	185 (6)	
PA-VSD	11 (5.5)	143 (5)	154 (5)	
Isolated aortic valve stenosis	5 (2.5)	138 (4.8)	143 (4.6)	
Isolated mitral valve abnormality	2 (1)	78 (2.7)	80 (2.6)	
Ebstein disease and other tricuspid valve abnormalities	6 (3)	58 (2)	64 (2.1)	
Pulmonary valve stenosis	3 (1.5)	195 (2)	204 (6.6)	
Common arterial trunk	2 (1)	53 (1.8)	55 (1.8)	
Comorbidities, *n* (%)				
Acquired comorbidity	41 (20.3)	415 (14.4)	456 (14.8)	0.03
Congenital comorbidity	51 (25.3)	665 (23)	716 (23.2)	0.5
Severity of illness risk	38 (18.8)	336 (11.6)	374 (12.1)	0.005
Down’s syndrome	25 (12.4)	252 (8.7)	277 (9)	0.1
Additional cardiac risk factors	18 (8.9)	212 (7.3)	230 (7.4)	0.4
Prematurity	24 (11.9)	280 (9.7)	304 (9.8)	0.3
Any type of UVH, *n* (%)	68 (33.7)	356 (12)	414 (13.4)	<0.0001
Procedural data, *n* (%)				
Procedure type, *n* (%)				
Reparative/corrective	98 (48.5)	1627 (56.3)	1725 (55.8)	<0.0001
Palliative	71 (35.2)	439 (15.2)	510 (16.5)	
Ambiguous	33 (16.3)	822 (24.5)	855 (27.7)	
Bypass length, *n* (%)				
None	22 (10.9)	471 (16.3)	493 (16)	<0.0001
Up to 90 min	54 (26.7)	1244 (43.1)	1298 (42)	
90 min or over	126 (62.4)	1173 (40.6)	1299 (42)	
PRAiS score^a^ (%), median (IQR)	1.6 (0.6–3.7)	1.3 (0.6–3.5)	1.3 (00.6–3.5)	0.1
Postoperative specific morbidities, *n* (%)				
Necrotizing enterocolitis	11 (5.4)	64 (2.2)	75 (2.4)	0.009
Extracorporeal life support	19 (9.4)	43 (1.5)	62 (2)	<0.0001
Feeding issues	26 (12.9)	158 (5.5)	184 (6)	<0.0001
Renal support	29 (14.4)	114 (4)	143 (4.6)	<0.0001
Acute neurological event	14 (6.9)	52 (1.8)	66 (2.1)	<0.0001
Post surgical infection	19 (9.4)	66 (2.3)	85 (2.8)	<0.0001
Unplanned reintervention	30 (14.9)	116 (4)	146 (4.7)	<0.0001
Major adverse event	26 (12.9)	108 (3.7)	134 (4.3)	<0.0001

a Missing in *n* = 9 procedures.

CAVSD: complete atrioventricular septal defect; DORV: double outlet right ventricle; IQR: interquartile range; PA: pulmonary atresia; PPE/C: prolonged pleural effusion/chylothorax; PRAiS: Partial Risk Adjustment in Surgery Score; TGA: transposition of the great arteries; ToF: tetralogy of Fallot; UVH: univentricular heart; VSD: ventricular septal defect.

### Timing, location and severity of PPE/C

PPE/C occurred at a median of 6 days (interquartile range 3–10 days) after the index procedure (missing in *n* = 22). Location was documented in *n* = 179 cases of PPE/C. A right pulmonary PPE/C was seen in *n* = 72 (40.2%), a left pulmonary PPE/C in *n* = 51 (28.5%), a bilateral pulmonary PPE/C in *n* = 61 (34.1%) and a pericardial PPE/C in *n* = 12 (6.7%) of cases. No associations were found between patient/procedure factors and PPE/C location in univariable analysis.

Grading was documented in *n* = 181 of the total PPE/C: *n* = 3 were severe (1.7%), *n* = 32 were moderate (17.7%) and *n* = 146 were mild (80.6%). Among these, the only predictor for moderate or severe PPE/C in univariable analysis was severity of illness indicator (OR = 2.4, *P* = 0.05), but this also became not statistically significant after accounting for centre effect (*P* = 0.1). When compared to mild PPE/C, more than mild PPE/C was associated with longer HLoS (median 22 days vs 34 days, *P* = 0.01) and higher 6-month mortality (excluding ECLS: 5.2% vs 17.2%, *P* = 0.04). Early (30-day) mortality was not statistically different by PPE/C severity (excluding ECLS, mild: 0.7%, more than mild: 3.5%, *P* = 0.3), albeit there were only 2 events.

### PPE/C by index procedure type

Incidence of PPE/C in specific procedure types ranged from 0% to 33.3% (Table 2). Among the groups of procedures with more than 20 cases, the Fontan procedure was associated with the highest incidence of PPE/C (33.3%). Other procedures were associated with either moderate (10–15%) or low (5–10%) incidence of PPE/C (Table 2).

**Table 2:** Incidence of PPE/C, by main index specific procedure groups

	With PPE/C, *n* (%)	Total
High incidence (>15%)		
Fontan procedure	45 (33.3)	135
Moderate incidence (10–15%)		
Mitral valve replacement	3 (13.6)	22
CAVSD repair	16 (13.1)	122
Vascular ring procedure	11 (12.6)	87
Tricuspid valve repair	4 (11.8)	34
Arterial switch + VSD closure	4 (11.1)	36
Bidirectional cavopulmonary shunt	16 (11)	146
Tetralogy and Fallot type DORV repair	22 (10.4)	212
Low incidence (5–10%)		
TAPVC repair	4 (9.3)	43
Norwood procedure	6 (8.2)	73
Mitral valve repair	2 (8)	25
Arterial switch (for isolated TGA)	6 (7.1)	85
Arterial shunt	3 (6)	50
Very low incidence (5–10%)		
Ross operation	1 (3.5)	29
Partial AVSD repair	2 (3.3)	60
Isolated coarctation/hypoplastic arch repair	4 (3)	134
Isolated RV-PA conduit construction	2 (3)	66
Isolated pulmonary artery band	2 (2.1)	97
Subaortic stenosis repair	2 (2.1)	96
Cardiac conduit replacement	1 (2.1)	48
VSD repair	3 (1.4)	212
Aortic valve repair	1 (1.4)	74
ASD repair	0 (0)	125
Pulmonary valve replacement	0 (0)	51
Surgical PDA ligation	0 (0)	45
Sinus venosus ASD or PAPVC repair	0 (0)	35
Supravalvar aortic stenosis repair	0 (0)	25

Only index procedure groups with more than 20 total cases shown.

ASD: atrial septal defect; CAVSD: complete atrioventricular septal defect; DORV: double outlet right ventricle; PAPVC: partially anomalous pulmonary venous connection; PDA: patent arterial duct; PPE/C: prolonged pleural effusion/chylothorax; RV-PA: right ventricle to pulmonary artery; TAPVC: totally anomalous pulmonary venous connection; TGA: transposition of great arteries; VSD: ventricular septal defect.

### Factors associated with PPE/C

Predictors of PPE/C included Fontan type procedure (OR = 11.3, *P* < 0.0001), severity of the diagnosis group (OR up to 2.9 compared to least severe), and more than 90 min on bypass (OR = 1.8, *P* = 0.02, compared to no bypass). Table 3 details all factors associated with PPE/C, based on univariable and multivariable analysis.

**Table 3:** Factors associated with PPE/C in univariable and multivariable logistic regression

	Univariable analysis			Multivariable analysis		
	OR	95% CI	*P*-value	OR	95% CI	*P*-value
Weight	0.98/kg	0.97–0.99	0.04	0.98/kg	0.96–0.99	0.002
Patient sex (female)	0.8	0.68–0.94	0.005			
Diagnosis complexity						
A (most severe/complex)	6.4	5.5–7.6	<0.0001	2.9	1.8–4.7	<0.0001
B	5.4	4–7.4	<0.0001	2.2	1.2–4.1	0.009
C	4.1	2.8–6.1	<0.0001	2.7	1.5–4.9	0.001
D	3.9	3.3–4.6	<0.0001	2.6	1.4–4.8	0.002
E (least severe/complex)	Baseline	Baseline	Baseline	Baseline	Baseline	Baseline
PRAiS score (%)	1.02/%	1.002–1.03	0.03			
Acquired comorbidity	1.5	1.2–1.9	0.001	1.5	1–2.1	0.05
Down syndrome	1.5	1.03–2.3	0.04			
Prematurity	1.3	1.1–1.5	0.009			
Severity of illness risk	1.6	1.2–2.1	0.001			
Procedure type						
Reparative/corrective	Baseline	Baseline	Baseline			
Palliation	2.5	1.8–3.5	<0.0001			
Ambiguous	0.7	0.4–1.1	0.1			
Fontan procedure	8.6	4.9–15.2	<0.0001	11.3	6.8–19	<0.0001
Bypass duration						
None	Baseline	Baseline	Baseline	Baseline	Baseline	Baseline
Up to 90 min	0.9	0.4–2.2	0.9	0.7	0.4–1.2	0.2
90 min or more	2.3	1.1–5	0.04	1.8	1.1–3	0.01
Sternotomy sequence	1.4/additional resternotomy	1.2–1.6	<0.0001			

Only variables with a *P* < 0.1 in univariable analysis are shown. Values are from mixed model with centre random effect (intercept).

CI: confidence interval; OR: odds ratio; PPE/C: prolonged pleural effusion or chylothorax; PRAiS: Partial Risk Adjustment in Surgery.

When looking specifically at the subgroup undergoing the Fontan operation (*n* = 135), there were *n* = 45 cases of PPE/C (33%), 35 of which were isolated PPE/C. The HLoS was significantly longer in patients with Fontan and PPE/C, compared to no PPE/C, at a median 21 vs 11 days (*P* = 0.0001), but 30-day and 6-month mortality were similarly low in both groups (30-day: 0% vs 1.1%, *P* = 0.5, no 6-month deaths).

### Relationship between PPE/C, other postoperative morbidities and short-term outcomes

Because PPE/C appears highly associated with other postoperative morbidities, we sought to evaluate the early mortality (30-day and 6-month) and HLoS associated to PPE/C in this populations. Overall, the 30-day and 6-month mortality with PPE/C were 3% and 9.4%, respectively compared to 1.3% and 2.7% without.

From least, to most severe combination of morbidities (Supplementary Material, Table S4), there were *n* = 2419 (78.3%) cases with no morbidities, *n* = 111 (3.4%) with isolated PPE/C, *n* = 304 (9.8%) with single non-PPE/C morbidity, *n* = 47 (1.5%) with single morbidity + PPE/C, *n* = 121 (3.9%) with non-PPE/C multimorbidity, *n* = 26 (0.8%) with PPE/C multimorbidity and *n* = 62 (2%) with ECLS morbidity (of which *n* = 18 had associated PPE/C).

**Figure 1:**
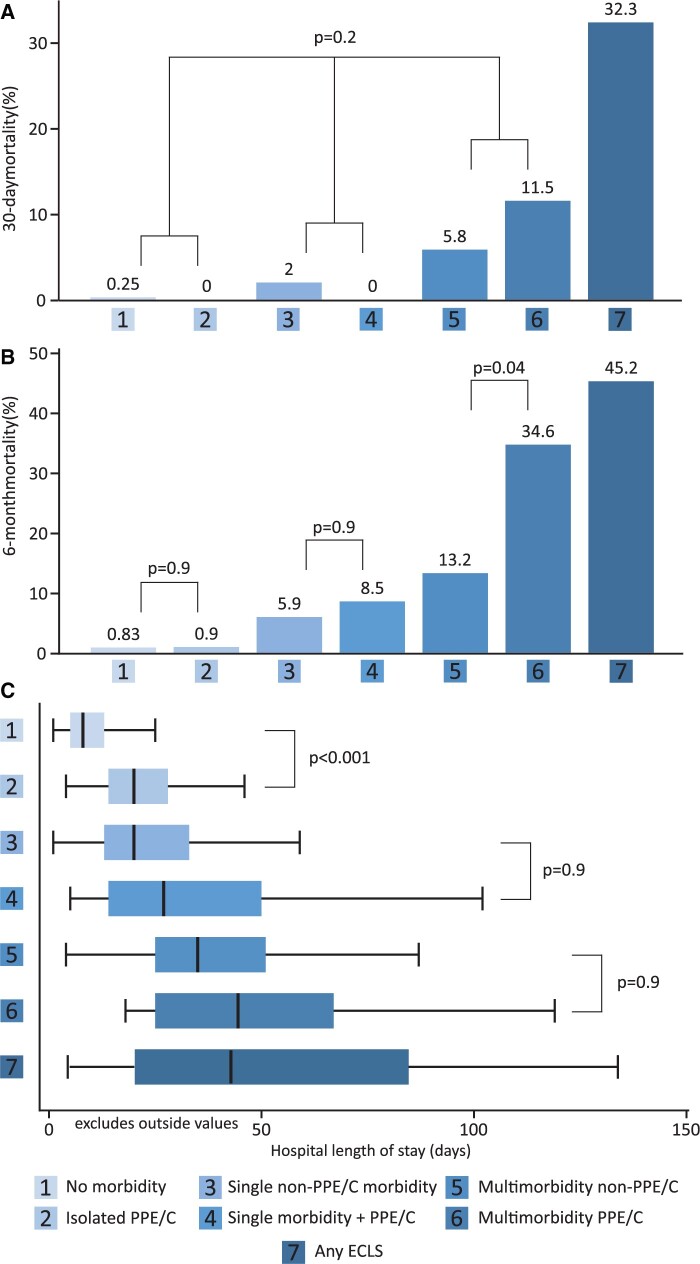
Impact of prolonged pleural effusion/chylothorax (PPE/C) on early outcomes after congenital heart disease surgery, by association with other post-operative morbidities. (**A**) 30-day mortality, (**B**) 6-month mortality and (**C**) hospital length of stay (HLoS). ECLS, extracorporeal circulatory life support. For panels (**A**) and (**B**), *P* values are from time-varying multiple-records survival analysis. For panel (**C**), *P* values are from linear mixed models for the whole cohort (same *P* values for analysis restricted to discharged alive).

Early (30-day and 6-month) mortality by morbidity subgroup is summarized in Fig. 1A and 1B. For 30-day mortality, the aggregate effect of PPE/C across all 3 associated morbidity groups was reported, without within-group pairwise comparisons, due to few events. Compared to no postoperative morbidity and single non-PPE/C morbidity, addition of PPE/C did was not associated with increased 30-day (0% vs 0.25%, and 0% vs 2%, respectively) or 6-month mortality (0.9% vs 0.83%, *P* = 0.9 and 8.5% vs 5.9%, *P* = 0.9, respectively). In multimorbidity scenarios, addition of PPE/C was associated with higher 6-month mortality (34.6% vs 13.2%, *P* = 0.04). The 30-day mortality was not statistically higher when PPE/C was present in multimorbid scenarios (11.5% vs 5.8%), but this comparison was underpowered and should be interpreted with caution. Proportion of different non-PPE/C comorbidities was similar between the PPE/C non-PPE/C pairs, with the exception of a slightly higher proportion of renal replacement therapy and unplanned reoperations in the PPE/C multimorbidity group, possibly driving part of the excess mortality. There were only *n* = 18 patients with both ECLS and PPE/C morbidities, with 30-day and 6-month mortality that were not statistically higher than ECLS without PPE/C (16.7% vs 38.6%, *P* = 0.1 and 27.8% vs 52.3%, *P* = 0.08, respectively).

HLoS of the index procedure by the presence of PPE/C morbidity is shown in Fig. 1C. Isolated PPE/C had longer HLoS compared to no morbidity, at 20 (14–28) vs 8 (5–13) days (*P* < 0.001 for whole group and hospital discharge survivors). Within the subgroups of single and multimorbidity, addition of PPE/C did not significantly increase HLoS, but the average effect of PPE/C across the whole population was an added 8 days of HLoS (*P* = 0.01) and 7 days for hospital survivors (*P* = 0.02), both excluding ECLS. The effect of added PPE/C decreased from those with no other morbidity, to 5 and 3 days in other single and multimorbidity, respectively. ECLS with PPE/C had longer HLoS compared to ECLS without PPE/C, at 83 (41–184) vs 30 (16–75) days, *P* < 0.001.

## DISCUSSION

In a large, prospective, multicentre study of unselected paediatric cardiac surgery cases, the incidence of PPE/C was 6.5%, with Fontan procedures, more complex anatomical diagnoses, neonatal age and longer cardiopulmonary bypass being independent risk factors. Having a PPE/C was highly associated with other post-operative complications, and these associations also influenced the impact of PPE/C on short term outcomes (Graphical Abstract). In simple scenarios, with either isolated PPE/C or at most another single morbidity, the presence of PPE/C does not negatively impact mortality, and only leads to prolonged hospitalization compared to cases with no complications. Within more complex scenarios, such as >2 morbidities, there appears to be an excess 6-months mortality associated with PPE/C, but it is difficult to disentangle the causal relations between multiple post-operative complications. For this reason, PPE/C should not be managed in isolation of other post-operative complications, with prevention and early diagnosis being likely the key to reducing the added burden on mortality, regardless of the direction of causality.

The issue of ‘true’ incidence of PPE/C has been discussed in the literature before, with various reports sharing various figures, ranging from 1.4% to 16.8%. While the core clinician definition of what constitutes a pleural effusion, or chylothorax are quite established, details such as clinical severity, size, biochemical composition and duration warranting flagging the event in hospital records vary greatly. More so, except for CHD surgery registries, single centre cohorts will always to susceptible to case mix bias. Based on a single, study-wide definition, we report an incidence of 6.5%, for a non-selected large cohort of patients undergoing paediatric cardiac surgery. Nevertheless, even using a clear definition, a complication such as PPE/C is prone to subjective interpretation of clinical characteristics, and we encourage central guidelines and definitions to be established to allow for more uniform reporting, before using PPE/C as a metric for quality of care.

This complex relationship between PPE/C and excess mortality and morbidity has proven difficult to assess in the past, with most reports being retrospective in nature. Early, or in-hospital mortality associated with PPE/C, or in most studies, chylothorax, ranged from 0% to 23% [3, 4, 6, 17, 18], smaller in multicentre or registry reports, likely owning to less selection bias, and more uniform definitions. Within our data, 30-day mortality for those with PPE/C was 3%, which compares favourably with existing literature, likely due to the prospective nature of the study allowing for complete ascertaining of events, especially in isolated, less clinically significant PPE/C, which might be missed in hospital record documentation and would be associated with lower mortality. This is supported by our reported incidence of 6.5%, which sits at the higher end of that described in other large multicentre registries [1, 3].

The relationship between PPE/C and excess early mortality has been investigated before, with mixed results. In the largest available study to date, including a total of 77 777 congenital heart surgeries, PPE/C was associated with a 2.13 higher risk of in-hospital death, compared to no PPE/C [4], with similar excess mortality found in other cohorts [3, 17]. On the other hand, in *n* = 1257 cases, Chan *et al.* [7] found no excess mortality associated with PPE/C, and Czobor *et al.* [19], using a propensity score matching approach did not show higher mortality with PPE/C. A major confounder, in addition to differences in case mix and event definitions, was adjusting for coexisting complications.

Our multicentre prospective design, aimed specifically at collecting post-operative morbidity data, provided enough information to allow for a more detailed analysis of true PPE/C-associated excess mortality. We found that having a PPE/C within a simple post-operative course tends to not be associated with excess mortality, but rather with prolonged HLoS. On the other hand, when PPE/C complicates an already existing multimorbidity scenario, it increases short-term mortality, more clearly at 6 months than at 30 days, while not affecting HLoS significantly. Interestingly, despite an association with ECLS, also described previously [3], PPE/C did not increase ECLS related mortality in our cohort. This is likely not due to the direct effects of PPE/C in severe or critical care cases, but the entangled effect of multimorbidity, where morbidity begets morbidity, and subsequent mortality. Based on these findings, active surveillance, prevention, and early intervention for PPE/C in cases with other existing post-operative morbidities could significantly impact early mortality [20]. In isolated PPE/C cases, reduction of hospital stays and prevention of secondary complications such as thrombosis or infection [20, 21], would allow for maintaining a low risk profile, where mortality is comparable to those without post-operative morbidities.

We found that several risk factors were associated with development of PPE/C, and these are in line with previous reports. The most clinically important predictor was undergoing the Fontan operation, and this was expected, with several reports in the literature describing high incidence of pleural effusion in this group of patients [22]. Despite this, the early mortality was very low, 0.7% at 6 months, unaffected by the presence of PPE/C, while HLoS was below the whole cohort average, making it unlikely for the effect of PPE/C on either outcome to be due to confounding with undergoing a Fontan procedure. Usually, PPE/C is reported to be associated mainly to single ventricle palliation and Tetralogy of Fallot surgery [1, 11, 17], but our data show that other commonly performed procedures have similar risks of PPE/C, either those of complex technical nature, or resulting in potential increases in venous pressure postoperatively, or both. This supports the hypothesis that postoperative PPE/C has multifactorial causes, rather than being a consequence of just a certain type of postoperative haemodynamic conditions increasing.

### Limitations

This study included consecutive patients with any type of CHD, undergoing any type of surgical procedure, and thus resulted in a high degree of patient diversity. In a minority of cases, the correct age at diagnosis of PPE/C, the location or severity were not documented, and due to the difficult conditions of the global pandemic, retrieval of missing data was not always possible at all centres. Biochemical nature of the PPE/C was not always documented, so analysis for chylothorax-only events was not attempted due to risk of misclassification. Postoperative antithrombotic protocols, timing of drainage tube extraction were not collected, and might have different by centre, or within each centre by era or team, possibly impacting the results, although all analyses did take into consideration centre-related unknown biases. At this point, an analysis on the temporal relationship between PPE/C and other morbidities, and a more detailed analysis of the impact of morbidity sequence on mortality was not attempted, as it is part of a future separate analysis of multimorbidity cases. Even with clear prospective definitions and surveillance, the timing of post-operative morbidities could not be ascertained correctly in 10–15% of patients, introducing unavoidable survivor bias and error in assessing exposure timing in these cases. Due to the small number of early deaths before exposure, we nevertheless do not judge the impact of this on the interpretation of the results to be notable. While the Fontan operation subgroup is of special clinical importance and identified as such within this project, it was not the aim of the study to investigate this, limiting the amount of disease-specific data of interest being collected and analysed.

## Conclusion

PPE/C is not uncommon after paediatric cardiac surgery, especially in the Fontan operation, complex disease or prolonged bypass duration. When occurring as an isolated complication or with at most one other morbidity, it is associated with a prolonged hospitalization, but not higher mortality. When associated to multimorbidity scenarios, the added presence of PPE/C appears to negatively impact mortality, but not HLoS. Early diagnosis of coexisting complications to prevent multimorbidity, and early management of multimorbidity PPE/C when it occurs should be implemented to reduce postoperative mortality.

## Supplementary Material

ezae363_Supplementary_Data

## Data Availability

The data underlying this article cannot be shared publicly due to ethical reasons underlying the privacy of individuals that participated in the study. Data can be shared on reasonable request to the corresponding author, within the limits of the laws and regulations governing the use of sensitive information.

## Notes

Presented at the 2023 Annual Meeting of the European Association for Cardio-Thoracic Surgery, Vienna, Austria (4-7 October 2023).

## ABBREVIATIONS

ECLS: Extracorporeal life-support
HLoS: Hospital length of stay
NCHDA: National Congenital Heart Disease Audit
PPE/C: Prolonged pleural effusion/chylothorax
PRAiS: Partial Risk Adjustment in Surgery Score
