# Supplementary material for: Risk factors and early outcomes associated with prolonged pleural effusion/chylothorax after paediatric cardiac surgery
Source: Eur J Cardiothorac Surg. 2024 Oct 7;66(4):ezae363. doi: 10.1093/ejcts/ezae363 (PMC11522863; doi:10.1093/ejcts/ezae363)
Supplement: ezae363_Supplementary_Data [file ezae363_supplementary_data.docx]

**Supplementary material**

***Dorobantu et al, Risk factors and early outcomes associated with prolonged pleural effusion/chylothorax after paediatric cardiac surgery***

**Supplementary methodology**

***National Audit data***

The specific National Congenital Heart Disease Audit (NCHDA) diagnosis and procedure code mapping used in Partial Risk Adjustment in Surgery version 2 (PRAiS) score, is publicly available at https://xip.uclb.com/i/software/PRAiS.html, and used also in the paper by Brown et al.^1^ It consists of grouping based on anatomy, procedure details and estimated outcomes. Specific procedure code mapping consists of a list of 77 procedures, with grouping being allocated on a pre-defined algorithm that determines the main procedure. PRAiS diagnosis mapping consist of 11 groups, while procedure mapping of 16 groups (<https://xip.uclb.com/i/software/PRAiS.html>).^2^

Surgical and interventional procedure data are submitted by each centre in the United Kingdom and audited both internally and externally to ensure data quality. For the purpose of this study, the data submission copy held at each participating centre was obtained, after pseudo-anonymisation. Alongside procedure and diagnosis codes the following are also collected and were used to define covariates in this study: patient demographics (gender, age at hospital admission, age at procedure, age at discharge, weight at procedure, low weight using national Z scores), diagnosis severity group (as previously defined by Rogers et al.)^3^ and a set of patient comorbidities present at the time of the procedure (acquired comorbidity, congenital comorbidity, severity of illness risk, Down’s syndrome, additional cardiac risk factors and prematurity),^3,4^ procedure type (reparative/corrective, palliative or ambiguous), bypass duration. All of these variables are part of the PRAiS score and used routinely to assess procedural risk in children undergoing congenital heart disease surgery.

***Post-operative morbidity data collection***

The study population included patients less than 17 years undergoing cardiac surgery and open, closed, or hybrid procedures involving the heart at each of 5 participating. The patients was included between October 1, 2015, and June 30, 2017. Not included were premature babies undergoing persistent ductus arteriosus ligation, cardiac transplant or tracheal procedures.

A set of 9 morbidities were collected, based on previous definitions (Supplementary Table S1, reproduced from Brown et al.).^1^ These were unplanned reinterventions, extracorporeal life support (ECLS), necrotising enterocolitis, prolonged pleural effusion or chylothorax (PPE/C), renal replacement therapy, major adverse events, acute neurological events, feeding issues and postsurgical infection. Morbidities were documented prospectively by research staff at each centre and attributed to the procedure most closely preceding each. All but 30-day unplanned reintervention and mediastinitis were documented within the same hospitalization as the index procedure, while the former two could be identified outside the initial hospital stay.

Of interest for the current analysis, pleural effusion or chylothorax was defined as either a chylous pleural effusion or significant chylous pericardial effusion or significant chylous ascites or a prolonged nonchylous effusion. Prolonged pleural effusion was defined as necessitating thoracic drainage >10 days (i.e. effusion noted in patient records beyond 10th day postoperatively). Age at PPE/C was based on the time PPE/C was first mentioned in patients records or noted by the study nurse. Chylothorax could be noted at any point during hospital stay, regardless of duration, if documented through standard-of-care testing (Supplementary Table S1). This classification is described in Supplementary Figure S1 below. There were five encountered scenarios: 1. Effusion was noted post-operatively, including cases where present before the index procedure, but resolved before day 10 post-operatively. In this case this does not meet the definition of PPE and was not classified as PPE/C; 2. Effusion was noted and drained intermittently during the postoperative hospital stay, noted also beyond day 10. This was classified as PPE/C and age at PPE/C was first time reported, with chest drain redo details not being recorded; 3. Effusion was documented postoperatively and continuous beyond day 10. This was classified as PPE/C and age at PPE/C was at first time noted; 4. Effusion was documented after day 10 post-operatively at any time during the hospitalization for the index procedure. This was classified as PPE/C regardless of duration with age at PPE/C being at first time noted; 5. If chylothorax was documented based on biological criteria (Supplementary Table S1) at any time during the hospitalization, at any duration, this was classified as PPE/C and age at PPE/C was when effusion was first documented.


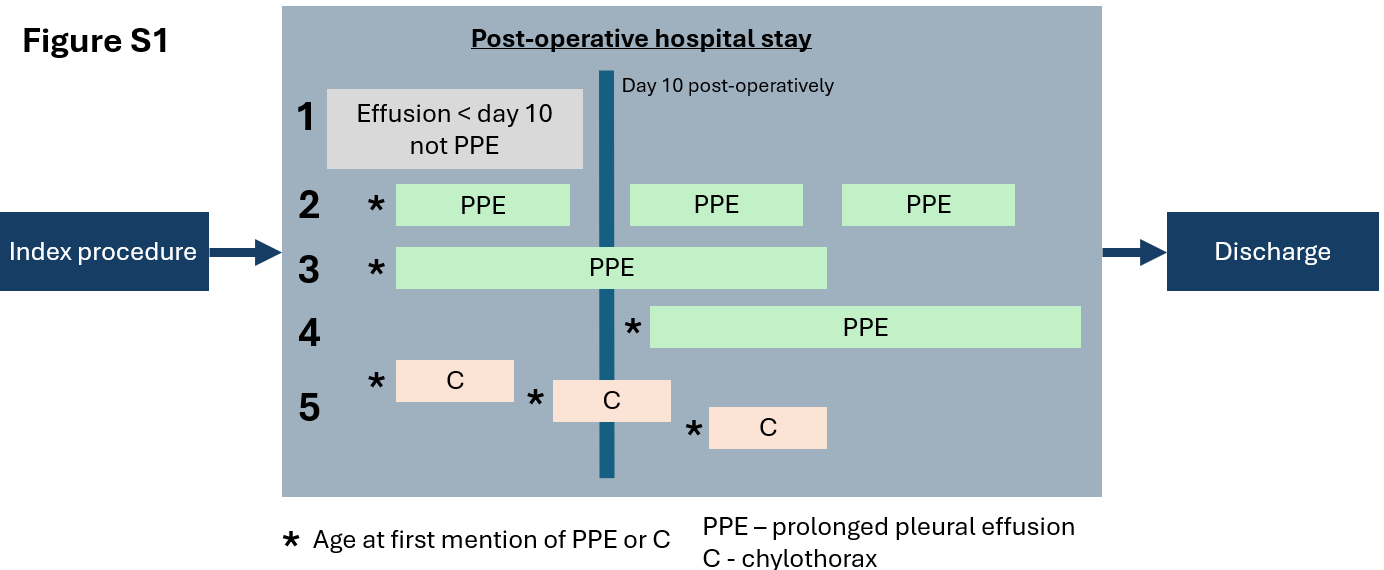


Accurate chylous vs non-chylous classification could not be obtained throughout, without additional testing, so this was not used in this analysis. PPE/C severity was defined based on volume of fluid loss as: mild – always <3 ml/kg/hour or average always <72 ml/kg in 24 hours; moderate – 3-10 ml/kg/hour or average 73-240 ml/kg in 24 hours; and severe – >10 ml/kg/hour or average >240 ml/kg/24 hours.

There were three steps taken to ensure data validation: monthly conference calls within the research team to ascertain any ambiguous cases; a 3-month sample of data from each study site being checked against the NCHDA for 5 of the 9 morbidities; and a final reconciliation at the end of the study at each centre for missing data. This allowed for complete data on outcomes of interest, with only missing data being on secondary characteristics such as age at morbidity or grading.

Life status was determined by a combination of NCHDA records and hospital patient records, ascertained at 6 months after the first procedure recorded for each patient. Hospital length of stay (HLoS) was calculated as the difference between age at admission and age at discharge from the cardiac centre (not including palliative care or secondary care admissions afterwards).

***Statistical analysis***

To identify factors associated with PPE/C (and moderate or severe PPE/C), we performed univariable and multivariable mixed models logistic regression analysis with PPE/C event (or PPE/C severity) as the dependent variable, centre as a random effect factor (intercept), and the following independent variables: age at procedure (continuous), neonatal age, weight (continuous), low weight for age, patient sex, diagnosis complexity group (ordinal), PRAiS score (continuous), acquired comorbidity, congenital comorbidity, prematurity, Down’s syndrome, severity of illness risk presence, additional cardiac risk factors, procedure type (palliation/corrective/ambiguous), undergoing a Fontan procedure (due to PPE/C being a common complication of this palliation route), bypass length (none/up to 90 min/90 min or more) and sternotomy sequence (from 0 to 6 or more, ordinal). A combination of stepwise forward selection and backward elimination was used. The final multivariable model was derived by fitting the model using all significant associated factors (p<0.05). initially all candidate variables were fitted, and at each step the variable with the highest p value (>0.05) was eliminated in a backward elimination manner, until all variables left were independently significant (p<0.05). Then, every variable with a p<0.1 in the univariable analysis was sequentially added to the model in a forward stepwise manner, and dropped if it remained statistically significant (p<0.05); at this step if any other variable in the model had a p<0.05, it was dropped (forward stepwise approach did not change the final model obtained in the backward elimination process). At each step the model log likelihood was evaluated. The final multivariable model was derived by fitting a logistic regression model for all significant associated factors (p<0.05). Only one factor was associated with severity of PPE/C so no multivariable model was obtained. Univariable analysis results for the candidate variables are shown in Supplementary Table S2 (PPE/C) and Supplementary Table S3 (moderate or severe PPE/C). Variance inflation factor was used to evaluate significant variable collinearity, which was not observed.

PPE/C can potentially be a direct consequence of the index procedure, but also of subsequent post-operative events, with exact causality being difficult to ascertain. As such, to investigate 30-day mortality, 6-moth mortality and HLoS attributable to the presence of PPE/C, we investigated the interaction between PPE and other post-operative morbidities. We defined an “associated postoperative morbidities” variable to be: “0” if no morbidities, “1” if one non-PPE morbidity, “2” if two or more, non-PPE, non-ECLS morbidities. ECLS was excluded as an associated morbidity for this comparison, due to the high impact on mortality and propensity for other major adverse events.10

To account for survivor and immortal time biases in 30-day and 6-month mortality analysis, a survival analysis with time-varying covariates in multiple-record data approach was used. For patients having documented PPE/C or first non-PPE/C morbidity (time varying covariates), an additional entry was included marking the time of status change, with the last entry containing the alive/dead status. If both PPE/C and first non-PPE/C morbidity occurred at the same time, only one status change was recorded, for both time varying covariates. In some patients, time to PPE/C (11%) or first non-PPE/C morbidity (15%) was not documented, and it was assumed that exposure occurred at first day for both time-varying covariates. For 30-day mortality, due to some substrata with no observed events preventing Cox regression, the aggregated effect of PPE/C was reported using log-rank testing on associated morbidity strata. For 6-month mortality time varying PPE/C status and associated postoperative morbidities were included as a full factorial interaction term in a multiple-record Cox regression, stratified by center. For the analysis of PPE/C on HLoS analysis, a linear regression mixed model (maximum likelihood estimation and Satterthwaite approximation of degrees of freedom with) was used, first on the whole cohort, then restricted to those discharged alive. PPE/C status and associated morbidity group were included as fixed interaction terms and center as random effects term. Effect of PPE/C on 6-month mortality and HLoS was reported by associated postoperative morbidities group using compared estimated margins within each associated morbidity stratum, using Bonferroni adjustment for multiple pairwise tests (3 within-family tests). These analyses were repeated including PRAiS score as a covariate, obtaining similar results. Mortality and HLoS data presented in main manuscript Figure 1 are calculated from the whole dataset (not estimated, including those dying within hospitalization for HLoS), while the p-values provided are obtained from the models described above (p values with and without discharged alive restriction for HLoS effect).

| **Supplementary Table S1:** Post-operative morbidities definitions (from Brown and colleagues).^1^ | | | | |
| --- | --- | --- | --- | --- |
| **Morbidity** | **Timescale for identification** | **Definition** | **Measurement protocol (if additional to definition)** | **Minimum treatment protocol** |
| **Acute Neurological Event** | Includes neurological morbidities that, based on best clinical judgement, arose as *new* findings around the time of surgery that were detected within the same hospitalisation as the surgery. It is recognised that in certain circumstances such as where a child is very sick on life support, pre procedure assessment is challenging, in these circumstances as full an evaluation as possible to be completed, incorporating serial assessments over time. | Neurological events including: seizure, abnormal movement (includes choreiform or athetoid), focal neurological deficit (includes hemiplegia and monoplegia), intracranial haemorrhage, stroke, brain death, Reversible Ischaemic Neurological Dysfunction, Hypoxic Ischaemic Encephalopathy, spinal cord ischaemia, basal ganglia damage or brain stem injury (includes abnormal cough or gag reflex). | Includes new abnormality in any of the following:  - Electroencephalogram.  - Brain scan (either computerised tomography or magnetic resonance).  - Clinical evaluation: Seizures or movement disorder, focal neurological signs, generalised neurological signs, altered conscious level including even brain death. | The treatment protocol is variable depending on the type of neuro-morbidity.  Specialist consultation with a neurologist, a full evaluation of any brain injury and neuro-developmental follow up would be a minimum. |
| **Unplanned reoperation or re-intervention** | Unplanned re-interventions are procedures outside the expected patient pathway, which may be undertaken at any time from the start of the post-operative admission up until 30 days following the primary operation. Additional procedures or revisions undertaken within the primary trip to the operating theatre (incorporating return onto cardiopulmonary bypass) are not included in the definition of re-operation. | Unplanned re-interventions include procedures that were not intended during the planning phase, follow an initial primary cardiac surgery and result in “substantive alteration to heart” incorporating cardiac bypass, cardiac non bypass, pacemaker placement, interventional catheterisations and also diaphragm plications (which are not related to the heart itself). The definition does not include support or other non-cardiac surgery procedures. | Unplanned return to the operating room or cardiac catheter laboratory within 30 days  (Excludes interventional catheters that were planned pre-operatively; excluding delayed chest closure, excluding procedures for bleeding)  (Includes diaphragm plication and insertion of pace maker for surgically acquired arrhythmia). | Not applicable. The minimal assessment is cardiovascular evaluation of the repair with echocardiography and tolerance of weaning from life supports. |
| **Feeding problems** | A diagnosis of post-operative feeding problems should be considered during recovery after surgery and prior to discharge from the specialist centre either to home or to secondary care if the child is unable to feed normally. The goal is detection of feeding problems which are new post-surgery, and it is recognised that this may be challenging where a child was not fed pre-operatively for cardiac reasons since feeding ability will not have been assessed objectively. | A child may fail to feed normally following paediatric cardiac surgery for a range of reasons including gastro-oesophageal reflux, vocal cord paralysis, oral-motor dysfunction, oral aversion, and neurologic impairment. If for any of these reasons a child is not able to orally feed or completely orally feed and is tube dependent at discharge from the tertiary centre or at 30 days (if he or she is otherwise clinically stable enough to feed at that time point), then a post-operative feeding problem will be diagnosed. | The requirement for any feeding support.  Includes via the intravenous route or via an enteral tube.  Excludes feeding support that was present to treat a primary problem diagnosed before the surgery, feeding support related to an episode of necrotising enterocolitis, and feeding support because the child dislikes a special diet. | Treatment includes assessment by the dietician, speech and language therapist and of the patient’s weight. Progress with feeding should be monitored by the clinical care team responsible at each stage of the journey. |
| **Need for renal replacement therapy** | Includes renal replacement therapy when initiated as a new support at any time from the start of the post-operative admission to ICU up until 30 days following the primary operation. | The child requires renal replacement therapy (peritoneal dialysis or haemofiltration) for renal failure (oligo-anuria of less than 0.5 ml/kg/hour and elevated creatinine level for age) and or fluid overload. In patients where renal support is required alongside extracorporeal life support, the primary morbidity is viewed as extracorporeal life support. | The measurement protocol is simply the presence of (new) renal support. (Excludes renal support on extracorporeal life support). Data on renal biochemistry and urine output will be collected. | Instigation of effective renal replacement therapy.  If recovery of kidney function does not occur within 3 to 5 weeks then consultation with paediatric renal physician is required. |
| **Major adverse cardiac events or never events** | Events within this morbidity may be identified during the tertiary hospital stay, either ward or intensive care unit (ICU), following the primary surgery. | These morbidity includes:  - Cardiac arrest, where the child receives any chest compressions or defibrillation.  - Chest re-opening on the ICU or ward for any reason.  - Major haemorrhage in the ICU following surgery.  - A ‘Never Event’ applicable to paediatric cardiac surgery defined as part of the United Kingdom National Health System (NHS) protocols.These include: wrong site or wrong patient surgery, wrong prosthesis surgery, retained foreign object post procedure, wrong route administration of medication, transfusion or transplantation of main red cell group incompatible blood components or organs, misplaced naso-gastric or oro-gastric tubes, tissue injury to limb or vital organ such as perforated viscus or ischaemic limb injury. | Major haemorrhage is defined as bleeding > 10ml/kg/hr on ICU for 2 consecutive hours.  A ‘Never Event’ includes the events listed plus harm to the patient, for example: if a naso-gastric tube is misplaced, detected and removed in a timely manner before any harm is done then this is not a ‘Never Event’. Conversely, if the misplaced naso-gastric tube is not noted, and feed is given into the bronchus, then this is a ‘Never Event’. | All events will results in immediate treatment as part of current practice. |
| **Extracorporeal life support** | Extracorporeal life support following surgery and before discharge from the tertiary hospital, including the rare cases when a child was on extracorporeal life support before surgery. | This morbidity is defined by the presence of an extracorporeal life support system connected to the patient following the operation, whether it was placed in the operating theatre or in the intensive care unit, and whether the indication was cardiac arrest, low cardiac output state, poor cardiac function, arrhythmia, residual or recurrent cardiac lesion, pulmonary including pulmonary hypertension or sepsis. | It is recognised that children on extracorporeal life support following paediatric cardiac surgery have high rates of other complications including renal support, bleeding, sepsis, sternal reopening, and cardiac arrest. Where such complications arise as part of extracorporeal life support, the morbidity is defined as extracorporeal life support. | The morbidity is in fact a treatment modality offered so this is not applicable. Centres offering extracorporeal life support follow protocols based on those provided by the extracorporeal life support organisation. |
| **Necrotising enterocolitis** | Necrotising enterocolitis as a new diagnosis from after surgery until discharge from the tertiary hospital. | Necrotising enterocolitis class 1a or 1b, which incorporates babies with systemic signs of inflammation and abdominal clinical signs such as distension or larger than normal gastric aspirates or mild rectal bleeding but no radiological changes are included, if a general surgery specialist has seen the child and commenced a course of intravenous antibiotics and parenteral nutrition for five to seven days. Cases of severe necrotising enterocolitis with radiological signs systemic instability and bowel perforation are also included. | Data in respect of systemic clinical signs, intestinal signs and radiology will be collected, as well as the treatments deployed, thus enabling the necrotising enterocolitis diagnosis to be graded between 1a and 3b. | Consultation with general surgery and further management in respect of antibiotics, nutrition, radiological investigation and surgical intervention. |
| **Surgical site infection and bloodstream infection** | Surgical site and blood stream infections diagnosed within the hospital admission following surgery or following readmission to the same unit during post-operative recovery, where the treating clinical team assesses the infection to be linked to the recent operation. It is noted that mediastinitis may be detected more than 30 days after cardiac surgery hence this time cut off is not applicable. | Deep surgical site Infection and/or mediastinitis includes any infection of an incised wound that undergoes any re intervention by a surgeon (such as opening of the wound, vacuum dressing), mediastinitis and false aneurysm, independent of culture positivity.  Blood stream infection includes both catheter related and non-catheter related. Cases have systemic signs of infection, a positive culture not judged to be a contaminant, and in the case of line related a catheter in place with positive cultures from the line or from the line tip when removed.  Endocarditis based on clinical, imaging or culture evidence judged to be diagnostic of endothelial/endocardial infection and its sequelae cardiac or extra-cardiac. | Deep surgical site infection excludes superficial site infection managed without a surgeon’s reoperation by conventional nurse dressing only, even if the wound heals by secondary intention. | The minimum treatment protocol consists of antibiotics based on organism and sensitivities, and where relevant the removal of the line. Surgical intervention may be required for deep surgical site and in some cases of endocarditis. Both conditions require prolonged antibiotic therapy. |
| **Prolonged pleural effusion or chylothorax** | Prolonged pleural effusion is a post procedural effusion with duration greater than ten days. Chylothorax is diagnosed from after surgery until discharge from the tertiary hospital. | Either a chylous pleural effusion or significant chylous pericardial effusion or significant chylous ascites or a prolonged non-chylous effusion that necessitates thoracic drainage at least ten days following index cardiac surgery. | Chylous effusions are characterised by milky appearance and a pleural fluid white blood cell count of greater than 1000 cells/μl with lymphocytes greater than 80%. If the child is on normal feeds the triglyceride level in the pleural fluid will be > 1.1 mmol/L or the ratio between the pleural triglyceride level and the serum triglyceride level will exceed 1. | Diet consisting of medium chain triglycerides or low fat for chylothorax. On a patient-by-patient basis other treatments include parenteral nutrition, octreotide infusion, intervention for venous obstruction thoracic duct ligation, and pleuradhesis. |

| **Supplementary Table S2:** Univariable analysis of risk factors associated with PPE/C | | | |
| --- | --- | --- | --- |
|  | OR | 95% CI | p value |
| Age at procedure, years | 0.96/y | 0.91-1.02 | 0.2 |
| Neonate | 1.08 | 0.69-1.72 | 0.7 |
| Weight | 0.98/kg | 0.97-0.99 | 0.04 |
| Low weight for age | 0.92 | 0.68-1.22 | 0.6 |
| Patient sex (female) | 0.8 | 0.68-0.94 | 0.005 |
| Diagnosis complexity group |  |  |  |
| A (most severe/complex) | 6.4 | 5.5-7.6 | <0.0001 |
| B | 5.4 | 4-7.4 | <0.0001 |
| C | 4.1 | 2.8-6.1 | <0.0001 |
| D | 3.9 | 3.3-4.6 | <0.0001 |
| E (least severe/complex) | baseline | baseline | baseline |
| PRAiS score (%) | 1.02/% | 1.002-1.03 | 0.03 |
| Acquired comorbidity | 1.5 | 1.2-1.9 | 0.001 |
| Congenital comorbidity |  |  |  |
| Down syndrome | 1.5 | 1.03-2.3 | 0.04 |
| Prematurity | 1.3 | 1.1-1.5 | 0.009 |
| Severity of illness risk | 1.6 | 1.2-2.1 | 0.001 |
| Additional cardiac risk factor |  |  |  |
| Procedure type |  |  |  |
| Reparative/corrective | baseline | baseline | baseline |
| Palliation | 2.5 | 1.8-3.5 | <0.0001 |
| Ambiguous | 0.7 | 0.4-1.1 | 0.1 |
| Fontan procedure | 8.6 | 4.9-15.2 | <0.0001 |
| Bypass duration |  |  |  |
| None | baseline | baseline | baseline |
| Up to 90 min | 0.9 | 0.4-2.2 | 0.9 |
| 90 min or more | 2.3 | 1.1-5 | 0.04 |
| Sternotomy sequence (continuous) | 1.4/resternotomy | 1.2-1.6 | <0.0001 |
| The odds ratio (OR), 95% confidence intervals (CI) and p values are from mixed logistic regression models with PPE/C as dependent variable, candidate risk factors as fixed effects terms and centre as random effects term (intercept).  Factors significantly associated with PPE/C in univariable analysis and resulting multivariable model are presented in the main manuscript Table 3. | | | |

| **Supplementary Table S3:** Univariable analysis of risk factors associated with moderate or severe PPE/C | | | |
| --- | --- | --- | --- |
|  | OR | 95% CI | p value |
| Age at procedure, years | 0.96/y | 0.84-1.1 | 0.6 |
| Neonate | 2.13 | 0.61-7.47 | 0.2 |
| Weight | 0.99/kg | 0.94-1.04 | 0.7 |
| Low weight for age | 1.13 | 0.69-1.81 | 0.6 |
| Patient sex (female) | 1.45 | 0.88-2.41 | 0.1 |
| Diagnosis complexity group |  |  |  |
| A (most severe/complex) | 0.3 | 0.03-3.1 | 0.3 |
| B | 1.07 | 0.19-5.98 | 0.9 |
| C | 1.46 | 0.56-3.83 | 0.4 |
| D | 1.08 | 0.23-5.17 | 0.9 |
| E (least severe/complex) | baseline | baseline | baseline |
| PRAiS score (%) | 1.05/% | 0.99-1.12 | 0.06 |
| Acquired comorbidity | 0.64 | 0.25-1.64 | 0.4 |
| Congenital comorbidity | 1.06 | 0.61-1.87 | 0.8 |
| Down syndrome | 1.61 | 0.67-3.86 | 0.3 |
| Prematurity | 1.62 | 0.79-3.32 | 0.2 |
| Severity of illness risk | 2.51 | 0.83-7.55 | 0.1 |
| Additional cardiac risk factor | 0.3 | 0.04-2.4 | 0.3 |
| Procedure type |  |  |  |
| Reparative/corrective | baseline | baseline | baseline |
| Palliation | 0.75 | 0.32-1.78 | 0.5 |
| Ambiguous | 1.19 | 0.42-0.34 | 0.7 |
| Fontan procedure | 0.59 | 0.31-1.16 | 0.1 |
| Bypass duration |  |  |  |
| None | baseline | baseline | baseline |
| Up to 90 min | 0.26 | 0.1-0.66 | 0.005 |
| 90 min or more | 0.57 | 0.18-1.78 | 0.3 |
| Bypass duration (continuous) | 1.001 | 0.99-1.004 | 0.3 |
| Sternotomy sequence (continuous) | 0.84/resternotomy | 0.59-1.21 | 0.3 |
| The odds ratio (OR), 95% confidence intervals (CI) and p values are from mixed logistic regression models with moderate or severe PPE/C as dependent variable, candidate risk factors as fixed effects terms and centre as random effects term (intercept).  These results are based on the n=181 patients with PPE/C and documented severity grading. Combined moderate or severe PPE/C was the outcome, present in n=35 of patients. | | | |

| **Supplementary Table S4.** Definitions of subgroups based on presence of PPE/C and number of associated post-operative morbidities | |
| --- | --- |
| No morbidity | Neither PPE/C or any other pre-defined morbidity |
| Isolated PPE/C | Only PPE/C documented, no other pre-defined morbidity |
| Single non-PPE/C morbidity | Only one predefined morbidity observed, not a PPE/C and not ECLS |
| Single morbidity and PPE/C | PPE/C was observed and a single other pre-defined morbidity, excluding ECLS |
| Multimorbidity non-PPE/C | Two or more pre-defined morbidities, excluding PPE/C or ECLS |
| Multimorbidity PPE/C | PPE/C was observed and in addition, two or more pre-defined morbidities, excluding ECLS |
| Any ECLS | ECLS was observed, regardless of number or type of other pre-defined morbidities |
| The predefined post-operative morbidities are: unplanned reinterventions, extracorporeal life support (ECLS), necrotising enterocolitis, prolonged pericardial effusion/chylothorax (PPE/C), renal replacement therapy, major adverse events, acute neurological events, feeding issues and postsurgical infection (Supplementary Table S1) | |

**Supplementary Material References**

1. Brown KL, Ridout D, Pagel C, et al. Incidence and risk factors for important early morbidities associated with pediatric cardiac surgery in a UK population. In: *Journal of Thoracic and Cardiovascular Surgery*. Vol 158. Mosby Inc.; 2019:1185-1196.e7. doi:10.1016/j.jtcvs.2019.03.139

2. Jacobs JP, Franklin RCG, Béland MJ, et al. Nomenclature for Pediatric and Congenital Cardiac Care: Unification of Clinical and Administrative Nomenclature – The 2021 International Paediatric and Congenital Cardiac Code (IPCCC) and the Eleventh Revision of the International Classification of Diseases (ICD-11). *https://doi.org/101177/21501351211032919*. 2021;12(5):E1-E18. doi:10.1177/21501351211032919

3. Rogers L, Brown KL, Franklin RC, et al. Improving Risk Adjustment for Mortality After Pediatric Cardiac Surgery: The UK PRAiS2 Model. *Annals of Thoracic Surgery*. 2017;104(1):211-219. doi:10.1016/j.athoracsur.2016.12.014

4. Pagel C, Rogers L, Brown K, et al. Improving risk adjustment in the PRAiS (Partial Risk Adjustment in Surgery) model for mortality after paediatric cardiac surgery and improving public understanding of its use in monitoring outcomes. *Health Services and Delivery Research*. 2017;5(23):1-164. doi:10.3310/hsdr05230
